# The Influence of Lysosomal Stress on Dental Pulp Stem Cell-Derived Schwann Cells

**DOI:** 10.3390/biom14040405

**Published:** 2024-03-27

**Authors:** Karen Libberecht, Nathalie Dirkx, Tim Vangansewinkel, Wendy Vandendries, Ivo Lambrichts, Esther Wolfs

**Affiliations:** 1 Laboratory for Functional Imaging & Research on Stem Cells, Biomedical Research Institute (BIOMED), Faculty of Medicine and Life Sciences, Hasselt University, 3590 Diepenbeek, Belgium; karen.libberecht@uhasselt.be (K.L.); nathalie.dirkx@uhasselt.be (N.D.);; 2 VIB, Center for Brain & Disease Research, Laboratory of Neurobiology, 3000 Leuven, Belgium; 3 Laboratory for Histology and Regeneration, Biomedical Research Institute (BIOMED), Faculty of Medicine and Life Sciences, Hasselt University, 3590 Diepenbeek, Belgium; ivo.lambrichts@uhasselt.be

**Keywords:** Schwann cells, lysosomal stress, dental pulp stem cells, chloroquine

## Abstract

Background: Dysregulation of the endo-lysosomal–autophagy pathway has been identified as a critical factor in the pathology of various demyelinating neurodegenerative diseases, including peripheral neuropathies. This pathway plays a crucial role in transporting newly synthesized myelin proteins to the plasma membrane in myelinating Schwann cells, making these cells susceptible to lysosome-related dysfunctions. Nevertheless, the specific impact of lysosomal dysfunction in Schwann cells and its contribution to neurodegeneration remain poorly understood. Methods: We aim to mimic lysosomal dysfunction in Schwann cells using chloroquine, a lysosomal dysfunction inducer, and to monitor lysosomal leakiness, Schwann cell viability, and apoptosis over time. Additionally, due to the ethical and experimental issues associated with cell isolation and the culturing of human Schwann cells, we use human dental pulp stem cell-derived Schwann cells (DPSC-SCs) as a model in our study. Results: Chloroquine incubation boosts lysosomal presence as demonstrated by an increased Lysotracker signal. Further in-depth lysosomal analysis demonstrated an increased lysosomal size and permeability as illustrated by a TEM analysis and GAL3-LAMP1 staining. Moreover, an Alamar blue assay and Caspase-3 staining demonstrates a reduced viability and increased apoptosis, respectively. Conclusions: Our data indicate that prolonged lysosomal dysfunction leads to lysosomal permeability, reduced viability, and eventually apoptosis in human DPSC-SCs.

## 1. Introduction

Lysosomes are membrane-bound cytoplasmic organelles containing various enzymes involved in the breakdown and digestion of macromolecules such as proteins, nucleic acids, lipids, and carbohydrates [1]. The primary role of lysosomes is to break down and recycle cellular waste materials, damaged organelles, and foreign substances that enter the cell. This process is crucial for maintaining cellular homeostasis and preventing the accumulation of harmful substances. Lysosomes are often referred to as centers for recycling or garbage disposal within the cell [2,3]. Nevertheless, over the past years, research has indicated that lysosomes are involved in various cellular processes, including cell signaling, transcriptional processes, and many other functions [2,3,4]. 

Schwann cells and oligodendrocytes serve as the myelinating cells of the peripheral (PNS) and central nervous system (CNS), respectively, playing a critical role in maintaining a healthy nervous system by facilitating the conductance of action potentials [5,6]. During myelination, these cells exhibit an extremely high metabolic rate as myelinogenesis requires extensive levels of protein synthesis. This renders Schwann cells to be highly dependent on functional intact protein quality control systems including the lysosomal system [5,6]. Nevertheless, the precise impact of lysosomal dysfunction on myelinating cells and the ensuing neurodegeneration remains poorly understood, especially in the context of demyelinating peripheral neuropathies, where lysosomal dysfunctions have been reported before [5,7,8]. Lysosomal dysfunction in neurodegenerative diseases primarily arises from the gradual accumulation of misfolded and/or aggregated proteins, resulting from the overload and dysfunction of the lysosomal pathway [2,9]. Nevertheless, besides the toxic build-up of aggregated or misfolded proteins, the exact consequences at the lysosomal level are still largely unknown. 

When lysosomal function is impaired for an extended period, they enter a state known as lysosomal stress, during which lysosomes react to these dysfunctions [10]. Lysosomal stress is characterized by disruptions in the lysosomal pH, membrane integrity, and enzyme activity, resulting in cellular damage and the potential onset of diseases [10,11]. Recent findings have expanded the understanding of lysosomal stress, describing additional manifestations such as enlarged lysosomes, protein aggregation, increased reactive oxygen species, altered cation efflux, and the accumulation of LDL cholesterol. Hence, these features can be incorporated into the definition of lysosomal stress [10,12,13].

This study aims to elucidate the influence of prolonged lysosomal dysfunction on Schwann cells using chloroquine. Although chloroquine is an FDA-approved drug primarily used to prevent and treat malaria, recent findings have shown its ability to induce lysosomal dysfunction and stress in several cell types [14,15,16,17,18,19]. However, the influence of chloroquine and prolonged lysosomal dysfunction on Schwann cells has not yet been reported.

The isolation of human Schwann cells poses challenges due to ethical concerns and practical limitations, including the invasive techniques that can harm healthy tissue and low proliferation capacity in vitro [20,21]. Notably, Martens et al. successfully differentiated human dental pulp stem cells (DPSCs) into functional myelinating Schwann cells [22]. Since DPSCs are obtained from third molar extractions, considered medical waste with few ethical restrictions, they present an interesting stem cell source compared to other adult stem cell populations, such as bone marrow-derived mesenchymal stem cells [22,23]. Moreover, human DPSCs exhibit anti-inflammatory and immunomodulatory capacities, a high proliferative potential, and self-renewal abilities, and they retain their characteristics after cryopreservation [22,24,25]. These features make DPSC-derived Schwann cells an excellent model for studying human Schwann cell biology.

Our data demonstrate that prolonged lysosomal dysfunction on DPSC-derived Schwann cells induces lysosomal permeability, reduces cell viability, and eventually triggers Schwann cell apoptosis. Hence, by elucidating the impact of lysosome-induced dysfunction and stress on Schwann cells, our data may contribute to an enhanced comprehension of lysosomal dysregulation in peripheral neuropathies.

## 2. Materials and Methods

Cell culture—Ethical approval was obtained by the medical ethical committee of Ziekenhuis Oost-Limburg, Genk, Belgium (13/0104U). Following informed consent, third molars were collected from donors undergoing tooth extraction for orthodontic reasons. The dental pulp stem cells were isolated using the explant method as described previously [26] and cultured at 37 °C and 5% CO_2_ in minimal essential medium (αMEM, Thermo Fisher Scientific, Waltham, MA, USA), supplemented with 100 U/mL of P/S, 2 mM of L-glutamine, and 10% of fetal bovine serum (FBS, Biowest, Nuaillé, France). At low passage (2–3), the DPSCs were differentiated towards Schwann cells as previously described [22]. In brief, the DPSCs were grown for 24 h (h) in αMEM without FBS and supplemented with 1 mM of β-mercaptoethanol (Gibco, Thermo Fisher Scientific, Waltham, MA, USA) on a Poly-L-lysine-coated surface (Sigma-Aldrich, St. Louis, MO, USA). Hereafter, cells were incubated for 72 h with 35 ng/mL of trans-retinoic acid (Thermo Fisher Scientific, Waltham, MA, USA) in a standard αMEM supplemented with 5 µM of forskolin (Stemcell Technologies, Vancouver, BC, Canada), 10 ng/mL of basic fibroblast growth factor (Immunotools, Friesoythen, Germany), 5 ng/mL of platelet-derived growth factor AA (Immunotools, Friesoythen, Germany), and 200 ng/mL of Neuroregulin-1 (Immunotools, Friesoythen, Germany). The cells were maintained in this medium for at least 14 days, and DPSC-derived Schwann cells are further referred to as DPSC-SCs. Successful differentiations were verified using quantitative polymerase chain reaction (qPCR) and immunocytochemistry (ICC) to determine the expression levels of P75 neurotrophic receptor (P75^NTR^), S100 calcium-binding protein B (S100B), laminin, and myelin protein zero (MPZ).

Quantitative polymerase chain reaction—Cells were seeded on Poly-L-lysine-coated plates (Sigma-Aldrich, St. Louis, MO, USA) and allowed to attach overnight. Total mRNA was extracted using a QIAzol Lysis reagent (Qiagen, Hilden, Germany) according to the manufacturer’s protocol. Next, the mRNA quality was validated using a NanoDrop (Thermo Fisher Scientific, Waltham, MA, USA). Hereafter, 3 ng/μL of cDNA samples were synthesized using a T-100 Thermal Cycler (Bio-Rad, Hercules, CA, USA) and Qscript (Quantabio, Beverly, MA, USA). Eventually, qPCR SYBR Green master mixes (Thermo Fisher Scientific, Waltham, MA, USA), including primer pairs (*P75*: Fw 5′ AGTTGGACTGATTGTGGGTGT 3′, Rev: 5′ CAGGCACAAGGGCTTCTTTTT 3′, *S100b*: Fw 5′ AGGGAGACAAGCACAAGCTGAAGA 3′, Rev 5′ TGTCCACAACCTCCTGCTCTTTGA 3′, *laminin-alpha-2* (*LAMA2*): Fw 5′ TGAGTATGAAAGCAAGGCCAGA 3′, Rev 5′ TGGTAACACCAACATAATCGGG 3′, and *MPZ*: Fw 5′ GAGGAGGCTCAGTGCTATGG 3′, Rev 5′ TTCTGCTGTGGTCCAGCATT 3′), were added to the samples. Subsequently, a PCR cycle was used: 95 °C, 20″–[95 °C, 3″–60 °C, 30″]40× –95 °C, 15″–60 °C, 60″–95 °C, 15”. The primer efficiencies were validated before use. The fold changes (FC) were calculated from the Ct values and were normalized for a validated housekeeping gene (RefFinder software, https://www.ciidirsinaloa.com.mx/RefFinder-master/, (accessed on 1 December 2022), *phosphoglycerate kinase* (*PGK1*: Fw 5′ CTGGGCAAGGATGTTCTGTT 3′, Rev 5′ GCATCTTTTCCCTTCCCTTC 3′)).

Immunocytochemistry—The cells were seeded on Poly-L-lysine-coated glass coverslips at a density of 20,000 cells/well (Sigma-Aldrich, St. Louis, MO, USA). After reaching 70% confluency, the cells were washed with phosphate-buffered saline (PBS) and fixated for 20 min with 4% of paraformaldehyde (PFA) (Sigma-Aldrich, St. Louis, MO, USA). For the galactin3-LAMP1 assay, the cells were incubated with 20 µM of chloroquine (Sigma-Aldrich, St. Louis, MO, USA) for 5 h, washed with PBS, and fixated for 20 min with 4% of PFA. Next, the cells were washed with PBS and permeabilized for 15 min with 0.05% Tween-20 in PBS. Next, the cells were washed with PBS and blocked with 10% of serum-free protein block (Dako, Agilent, Santa Clara, CA, USA) for 1 h at RT. After overnight incubation at 4 °C with the primary antibody (P75^NTR^: Santacruz, sc-271708 1:400, S100B: Dako, Z0311, 1:300, MPZ: Abcam, ab31851, 1:250, Laminin1 + 2: Abcam, ab7463, 1:400, LAMP1: The Developmental studies hybridoma bank (DSHB), Iowa City, IA, USA, H3A4, 1:300; Galectin3, Abcam, Cambridge, UK, Ab190167, 1:300), the cells were washed and incubated with 1:300 secondary antibody (Alexa555, Abcam, Ab150074; Alexa488, Abcam, Ab150077) for 1 h at RT. Negative controls were included where the primary antibody was omitted. When indicated in the figure legends, the cells were washed and incubated for 20 min with Hoechst 33342 (ImmunoChemistry Technologies, Davis, CA, USA) at RT. Following a final washing step, the coverslips were mounted using an Immunomount (Thermo Fisher Scientific, Waltham, MA, USA). To evaluate the protein levels of several Schwann cell-related markers, images were acquired with a Leica DM4000 B LED microscope (20×). Next, the integrated density was calculated/cell using the Image J software (Rasband, W.S., ImageJ, U. S. National Institutes of Health, Bethesda, MD, USA, https://imagej.nih.gov/ij/, (accessed on 1 November 2019)). The data were normalized against undifferentiated DPSC control cells. For the galectin3-LAMP1 colocalization analysis, fluorescence images (Z-stacks) were acquired with the LSM880 (Zeiss) using a 63× objective (zoom 1) and AIRYscan feature. A colocalization analysis was performed by calculating the Pearson’s coefficient using the Image J software and Coloc2 plugin.

Live lysosomal imaging—The cells were seeded on a Poly-L-lysine-coated surface (Sigma-Aldrich, St. Louis, MO, USA) and allowed to attach. The cells were incubated with 20, 35, or 45 µM of chloroquine (Sigma-Aldrich, St. Louis, MO, USA) followed by a Lysotracker deep red (Thermo Fisher Scientific, Waltham, MA, USA). In brief, the cells were incubated with 20 nM of Lysotracker^®^ for 1 h at 37 °C and washed with PBS. Next, the cells were incubated with 20, 35, or 45 µM of chloroquine. The live cell imaging was performed at 37 °C and 5% CO_2_ using the Incucyte^®^ S3 Live Cell Analysis System (Sartorius, Gottingen, Germany). Images were made every 4 or 8 h using the standard scan settings with a 20× objective lens and were monitored for a period of 24 h. The Lysotracker data analysis was performed using the Incucyte^®^ S3 Live Cell Analysis System (Sartorius, Gottingen, Germany), by calculating the area (µm^2^) positive for the Lysotracker signal, and the data were normalized against manually counted cell numbers. Apoptotic cells were excluded from the analysis.

Cathepsin B activity assay—Cathepsin B (CtB) activity was monitored using the magic red assay, according to the manufacturer’s protocol (ImmunoChemistry Technologies, Davis, CA, USA). In brief, the DPSC-SCs were seeded at a density of 10,000 cells/cm^2^ in Poly-L-lysine-coated 8-well Ibidi chambers (Proxylab, Beloeil, Belgium) and incubated with 20 µM of chloroquine (Sigma-Aldrich, St. Louis, MO, USA) for 5 h. After washing with PBS, a new medium containing the magic red substrate was added and incubated for 1 h at 37 °C and 5% CO_2_. Next, the cells were incubated with Hoechst 33342 (ImmunoChemistry Technologies, Davis, CA, USA) in the medium for 15 min at 37 °C and 5% CO_2_, and visualized using confocal live imaging (Zeiss LSM880; Breda, The Netherlands). The magic red signal was measured at 594 nm with a 63× objective (zoom 1).

Alamar blue assay—The Alamar blue assay (Invitrogen^TM^, Thermo Fisher Scientific, Waltham, MA, USA) was performed according to the manufacturer’s instructions. In brief, 5000 cells were seeded in a Poly-L-lysine-coated 96-well plate and were allowed to attach overnight. Next, the cells were incubated with 20 µM of chloroquine (Sigma-Aldrich, St. Louis, MO, USA). Two, five, and twenty-four hours following incubation, the cells were washed with PBS, and 100 µL of Alamar blue solution (1:10 in medium, Sigma-Aldrich, St. Louis, MO, USA) was added to the cells and incubated for 4 h. The fluorescence was measured at ex/em 530/590 using a Fluostar Optima plate reader (BMG Labtech, Ortenberg, Germany). Readouts were performed 2, 5, and 24 h after administration of 20 µM of chloroquine (Sigma-Aldrich, St. Louis, MO, USA).

Propidium iodide assay—At 2, 5, and 24 h following 20 µM of chloroquine (Sigma-Aldrich, St. Louis, MO, USA) incubation, the cells were washed and lysed with Lysis reagent A100 (Chemometec, Lillerod, Denmark). Next, the cells were incubated in the dark with Propidium Iodide (PI) reagent (Chemometec, Lillerod, Denmark 1/50) for 15 min. The fluorescence intensity was measured ex/em 540/612 nm using a Fluostar Optima plate reader (BMG Labtech, Ortenberg, Germany).

Live caspase-3 imaging—The cells were seeded on Poly-L-lysine-coated plates (Sigma-Aldrich, St. Louis, MO, USA) and allowed to attach overnight. The cells were incubated with 20, 35, or 45 µM of chloroquine (Sigma-Aldrich, St. Louis, MO, USA) followed Caspase-3 staining (Sartorius, Gottingen, Germany), which was added according to the manufacture’s protocol. In brief, 5 µM of Caspase-3 dye was added to the medium, and the cells were incubated with 20, 35, or 45 µM of chloroquine. The live cell imaging was performed at 37 °C and 5% CO_2_ using the Incucyte^®^ S3 Live Cell Analysis System (Sartorius, Gottingen, Germany). Images were made every 4 h using the standard scan settings with a 20× objective lens and were monitored for 24 h. For analysis, the number of Caspase-3 positive and total cells were manually counted, and, based on this, the percentage of apoptotic cells were calculated. 

Transmission electron microscopy—The cells were seeded onto Poly-L-lysine-coated Thermanox^®^ slides (Thermo Fisher Scientific, Waltham, MA, USA), allowed to attach, and incubated with 20 µM of chloroquine (Sigma-Aldrich, St. Louis, MO, USA) for 5 h. Next, the cells were washed with PBS and fixated using 2% of glutaraldehyde in 0.05 M of cacodylate buffer (pH 7.3) at 4 °C. Then, the samples were post-fixed in 2% of osmium tetroxide in 0.05 m of sodium cacodylate buffer (a pH of 7.3) for 1 h. Subsequently, the cells were dehydrated in graded acetone concentrations and embedded in epoxy resin (Araldite; Basel, Switzerland). Ultra-thin sections (0.06 μm) were mounted on 0.7% formvar coated grids and contrasted with uranyl acetate followed by lead citrate. Samples were examined with a Philips EM 208 transmission electron microscope (Philips; Eindhoven, The Netherlands) operated at 80 kV. Images were obtained at a magnification of 11.000×.

Statistical analysis—The statistics were performed using the GraphPad Prism, version 9.3.1 (GraphPad Software, San Diego, CA, USA). Statistical outliers were removed for analysis using the Grubbs’ test (α = 0.05). Following the Shapiro–Wilk test for normality, the data were analyzed with the two-way ANOVA Dunnett tests, unpaired *t*-tests, or Mann–Whitney *t*-tests, as described for the specific experiments in the figure legends.

## 3. Results

### 3.1. DPSCs Successfully Differentiate towards Schwann Cells

**Figure 1:**
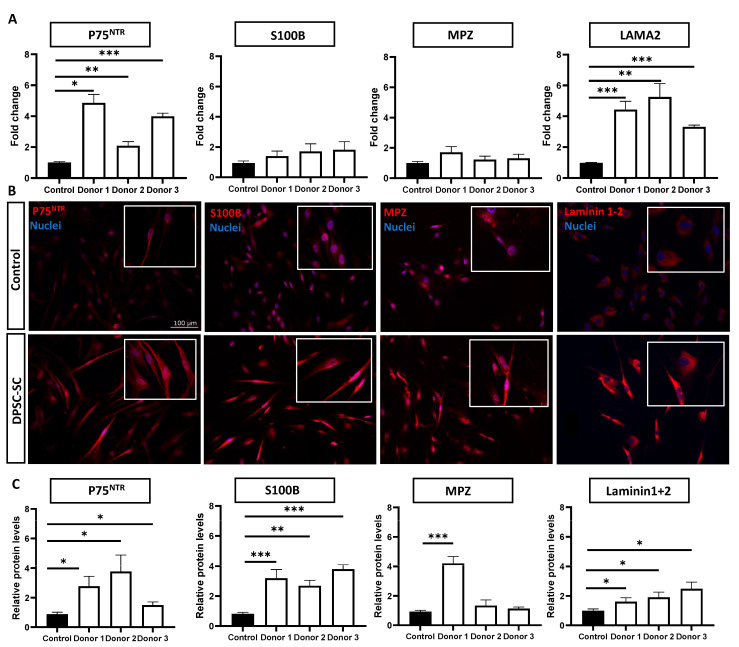
Increase in Schwann cell markers following DPSC-SC differentiation in three healthy donors. (**A**) Gene expression levels of *P75^NTR^*, *S100B*, *MPZ*, and *LAMA2* increased in all three donors. (**B**) Representative ICC images of P75^NTR^, S100B, MPZ, and laminin 1 + 2 before (control) and after DPSC-SC differentiation. (**C**) Quantifications of P75^NTR^, S100B, laminin 1 + 2, and MPZ ICC compared to undifferentiated controls. Hoechst staining was used to visualize nuclei. Data are represented as mean ± SEM. (**A**): *n* = three experiments with three technical replicates for each donor. (**C**): *n* = one experiment with three technical replicates for each donor. (**A**,**C**): Unpaired *t*-tests or Mann–Whitney test. Samples were compared towards undifferentiated DPSCs of the same donor. * *p* < 0.05, ** *p* < 0.001, and *** *p* < 0.0001. P75^NTR^: P75 neurotrophic receptor, S100B: S100 calcium-binding protein B, LAMA2: laminin alpha-2, and MPZ: myelin protein zero.

Three different DPSC donor lines were differentiated towards DPSC-derived Schwann cells, as previously described [22]. To confirm a successful differentiation, the Schwann cell-related marker expression was evaluated (Figure 1). We analyzed the gene expression levels of the Schwann cell markers *P75^NTR^* and *S100B* (Figure 1A). Both markers increased for all donor lines following the differentiation, and we observed a significant increase in *S100B* for donor 2, whereas *p75^NTR^* significantly increased in all donor lines. The gene expression levels of *MPZ*, the most abundant protein of the PNS myelin sheath [27], significantly increased in donor line 1, and increased (non-significantly) for donor lines 2 and 3. *LAMA2*, a key component of the Schwann cell basal lamina and crucial for Schwann cell differentiation [28,29], significantly increased in all lines. An ICC analysis confirmed an increase in Schwann cells-related markers at the protein level after the differentiation (Figure 1B,C). Both P75^NTR^ and S100B significantly increased after the differentiation in all donors. In addition, the MPZ levels increased, reaching significance in donor 1, and the laminin 1 + 2 levels significantly increased in all donor lines following the differentiation.

### 3.2. Chloroquine Induces Lysosomal Upregulation in DPSC-Derived Schwann Cells

**Figure 2:**
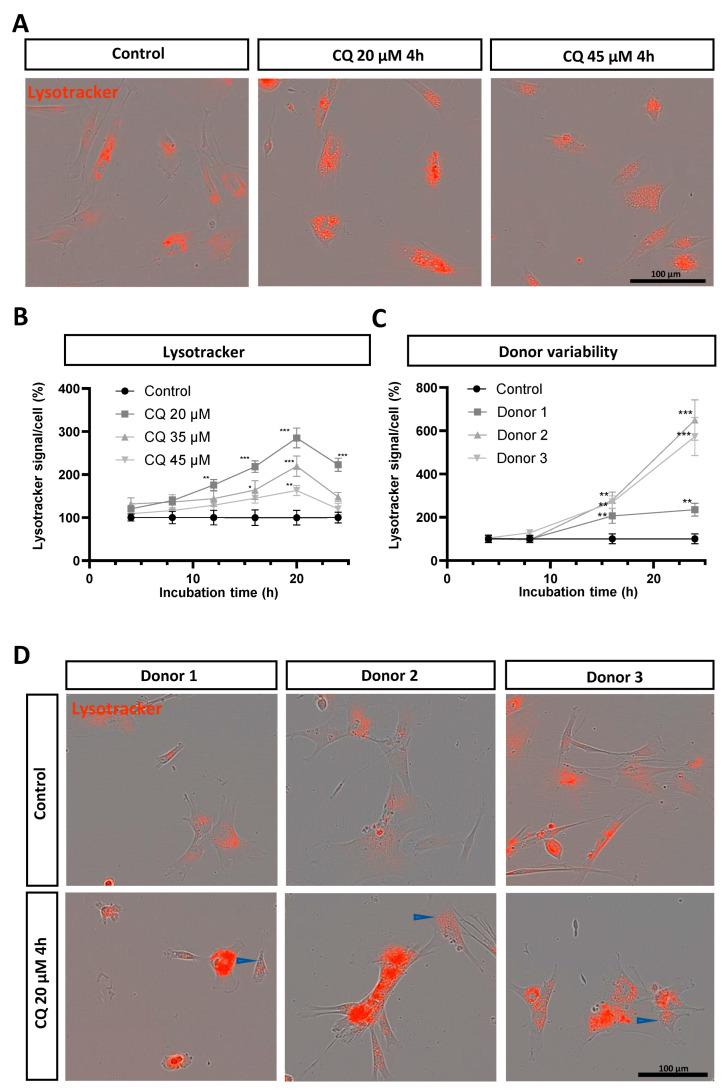
Increased Lysotracker signal after chloroquine stimulation in DPSC-SCs. (**A**) Representative images of chloroquine-stimulated vs. unstimulated control DPSC-SCs over time. (**B**) An increase in Lysotracker signal was observed for all three tested chloroquine concentrations (i.e., 20, 35, and 45 µM). DPSC-SCs incubated with 20 µM and 35 µM of chloroquine stimulation show a gradually increased Lysotracker signal, reaching significance starting from 12 h for 20 µM and 16 h for 35 µM, and remaining visible after 24 h (endpoint). At 20 h of chloroquine incubation, all monitored concentrations show a significantly higher Lysotracker signal compared to control cells. (**C**) Donor variability in Lysotracker signal after chloroquine stimulation compared to unstimulated control cells. All donor lines show significantly increased Lysotracker signal after 16 h of chloroquine incubation. (**D**) Representative images of chloroquine-stimulated vs. unstimulated control DPSC-SCs in three different donor lines. Blue arrows represent loss of Lysotracker fluorescence on the luminal side in a limited number of lysosomes. Data are presented as mean ± SEM. * *p* < 0.05, ** *p* < 0.001, and *** *p* < 0.0001. (**B**,**C**): Two-way ANOVA Dunnett test. (**C**): Chloroquine-exposed cells are compared to unstimulated control cells from the same donor line. *n* = one experiment with four to seven technical replicates. CQ: chloroquine.

To assess the response of Schwann cells to lysosomal overload, we exposed DPSC-SCs to chloroquine, a well-known inducer of lysosomal stress [10]. Different concentrations of chloroquine (20, 35, and 45 µM) were administered to the DPSC-SCs, and the Lysotracker fluorescence was monitored over time (Figure 2A,B). Our findings indicate a substantial increase in the Lysotracker signal across all conditions, compared to the unstimulated control cells (Figure 2B). Notably, 20 µM of chloroquine induced a significant increase in the Lysotracker signal starting from 12 h, peaking with a significant 1.9-fold increase at 20 h of stimulation, and a sustained increased after 24 h of stimulation. 

In contrast, 35 µM of chloroquine led to significantly increased fluorescence levels at 16 h and a 1.2-fold increase after 20 h of stimulation. At the same time, 45 µM of chloroquine induced a significant increase of the Lysotracker signal only at 20 h of incubation (Figure 2A,B).

Moreover, we assessed the effect of donor variability on lysosomal behavior upon chloroquine treatment over time using DPSC-SCs from three different human subjects (Figure 2C,D). The DPSC-SCs from all human donors showed increased Lysotracker signals after prolonged incubation with 20 µM of chloroquine. For all donors, we observed similar significant differences after 16 h and 24 h of stimulation with 20 µM of chloroquine (Figure 2C). For donor 1, however, it is important to note that already early on in the incubation step (starting after 4 h) we observed apoptotic DPSC-SCs, which was not seen in the other donor lines.

Upon chloroquine stimulation, an increase in lysosomal size was observed in all donors (Figure 2A,D). Furthermore, the intensity of the Lysotracker fluorescence gradually disappeared on the luminal side in a limited number of lysosomes, starting from 4 h of chloroquine stimulation, and this effect was observed in the cells from all human donors (Figure 2D, blue arrows). The loss of the Lysotracker signal in the lysosomal lumen increased over time with increasing chloroquine concentrations, and this effect is probably caused by changes in the lysosomal acid pH levels [30,31].

### 3.3. Chloroquine Induces Lysosomal Permeability in DPSC-Derived Schwann Cells

**Figure 3:**
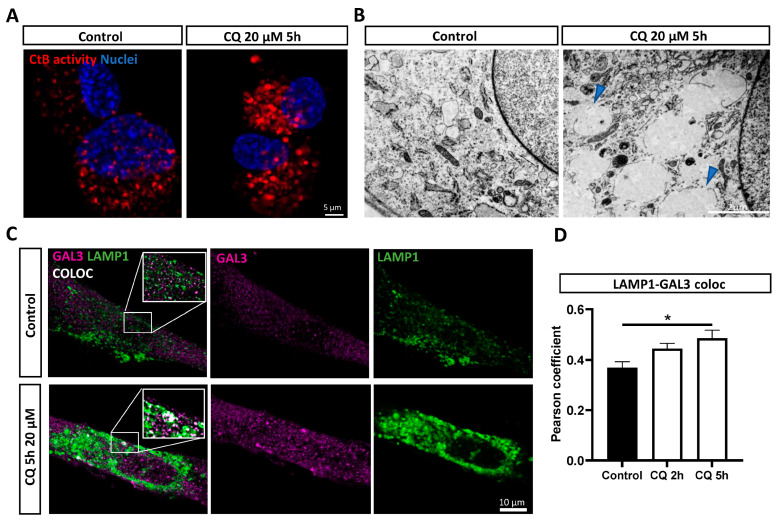
Increased lysosomal size and permeability in human DPSC-derived Schwann cells after chloroquine stimulation. (**A**) Representative images of Cathepsin B activity (red) illustrating an increased lysosomal diameter and CtB activity levels in chloroquine-exposed vs. unstimulated control cells. Hoechst staining was used to visualize nuclei. (**B**) TEM images showing enlarged, permeabilized lysosomes (blue arrows) after chloroquine stimulation in the cytosol of DPSC-SCs compared to unstimulated control cells. (**C**,**D**) A galectin 3-LAMP1 puncta assay confirmed a significant increase in the number of leaky or permeabilized lysosomes in human DPSC-SCs after 5 h of stimulation with 20 µM of chloroquine. Representative images (one optical Z section) are shown of the galectin 3-LAMP1 staining in chloroquine stimulated DPSC-SCs and unstimulated control cells in (**C**). * *p* < 0.05. (**D**): *n* = two individual experiments, with four to six technical replicates in each. Data are presented as mean ± SEM. Samples are compared to control cells using Mann–Whitney tests. CtB: Cathepsin B, LAMP1: Lysosomal associated membrane protein 1, Gal3: Galectin 3, and CQ: chloroquine.

An increased lysosomal size, swelling, and the loss of the Lysotracker signal have been linked to lysosomal permeability [30]. To further elucidate lysosomal changes, a magic red assay was performed, which showed an increase in the lysosomal size and cathepsin B activity after 5 h of 20 µM of chloroquine stimulation (Figure 3A). Subsequently, using a transmission electron microscopy (TEM), we confirmed the presence of enlarged and permeabilized lysosomes in the cytoplasm of chloroquine-exposed DPSC-SCs compared to unstimulated controls (Figure 3B, blue arrows). 

To further quantify the chloroquine-induced lysosomal permeability, we used a galectin 3 puncta assay. Galectin 3 acts as a sensor for leaky lysosomes by translocating towards the outer membrane in permeabilized lysosomes [30,32]. Hence, the colocalization of LAMP1 (a general marker for the lysosomal membrane) and galectin 3 is a marker for leaky lysosomes (Figure 3C,D). Our data show a significant increase in the Pearson’s correlation coefficient between the galectin 3 and LAMP1 expression in DPSC-SCs after 5 h of chloroquine incubation (R = 0.45) compared to the control cells (R = 0.34), confirming lysosomal permeabilization upon chloroquine-induced stress.

### 3.4. Reduced Viability and Apoptosis in Chloroquine-Exposed DPSC-Derived Schwann Cells

**Figure 4:**
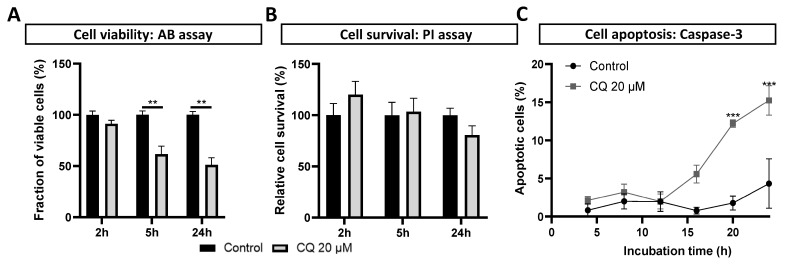
Reduced viability of human DPSC-Schwann cells after chloroquine stimulation. (**A**) Alamar blue assay showing significantly reduced viability in DPSC-derived Schwann cells, starting after 5 h of chloroquine stimulation, remaining observable even after 24 h. (**B**) Propidium iodide assay illustrating a non-significant decrease in cell survival after 24 h of chloroquine stimulation compared to control cells. (**C**) Caspase-3 live imaging, illustrating Schwann cell apoptosis starting from 20 h of chloroquine stimulation (20 µM). Data are presented as mean ± SEM. (**A**): *n* = Two individual experiments, with four technical replicates in each. (**B**): *n* = Three individual experiments with four technical replicates in each. (**C**): *n* = One individual experiment with four replicates. Samples were compared with controls at the same timepoint. (**A**): Mann–Whitney test, (**B**): Unpaired *t*-test, and (**C**): Two-way ANOVA Dunnett test. ** *p* < 0.001, and *** *p* < 0.0001. CQ: chloroquine, PI: Propidium Iodide assay, and AB: Alamar Blue assay.

Lysosomal permeability is often associated with lysosome-induced apoptosis [33]. Therefore, as a final step, we explored whether the chloroquine-induced lysosomal changes had an effect on the cellular health. To this end, an Alamar blue (AB) assay and a Propidium Iodide (PI) assay were performed following the chloroquine incubation to assess the impact of lysosomal stress on the Schwann cell viability and survival, respectively (Figure 4A,B). Our data show on average a significant decrease of 38% and 49% in viable and metabolic active human DPSC-SCs after 5 and 24 h of incubation with 20 µM of chloroquine, respectively (Figure 4A). However, using a PI assay, we observed no reduction in cell survival at 2 and 5 h of chloroquine administration (Figure 4B). However, a non-significant decrease in cell survival was observed after 24 h (a 19% decrease) in the chloroquine-incubated compared to the unstimulated control cells (Figure 4B)

We next monitored apoptosis using caspase-3 live imaging through the IncuCyte analysis, confirming no significant difference in apoptosis between the unstimulated control and chloroquine-stimulated human DPSC-SCs after 4, 8, 12, and 16 h of incubation (Figure 4C). Nevertheless, our data demonstrate significantly increased levels of caspase-3 and therefore apoptosis in DPSC-SCs after 20 h and 24 h of stimulation with 20 µM of chloroquine (a 12% and 15% decrease, respectively, Figure 4C).

## 4. Discussion

Schwann cells play a crucial role in the myelination process in the PNS. Their primary function is to wrap around axons, forming the myelin sheath, which is a specialized insulating layer facilitating the rapid conduction of nerve impulses along the axon [34]. During the process of myelinogenesis, Schwann cells exhibit a very high metabolic activity, necessitated by the substantial production of myelin lipids and proteins. This underscores their critical reliance on intact protein quality control mechanisms, including the lysosomal system [5,6,7,35]. Over the past decades, lysosomal dysfunction has been linked to several peripheral neuropathies; nevertheless, the exact consequences of lysosomal dysfunction in Schwann cells have not been elucidated [7,36]. At least partially, this is due to ethical and practical constraints in the isolation of human Schwann cells [20]. Hence, stem cell-differentiated Schwann cells are crucial to overcome these limitations [37]. Human dental pulp stem cells (DPSCs) offer a promising solution, as previous studies have successfully differentiated DPSCs towards functionally myelinating Schwann cells [22]. Additionally, DPSCs have a high proliferation capacity and can easily be cryopreserved even after differentiation. Furthermore, their use is associated with few ethical concerns, because they can be isolated from third molars, which are considered as medical waste [22]. Therefore, DPSC-derived Schwann cells (DPSC-SCs) provide an elegant approach for human in vitro modelling.

In this study, we differentiated three independent healthy donor lines towards Schwann cells. We observed a low basal expression of glial proteins in the DPSCs, in line with previous research illustrating a basal expression of Schwann cell and neuronal markers in DPSCs due to their neural crest origin [38]. Yet, following differentiation, the Schwann cell markers increased in all donor lines [38].

We aimed to elucidate the influence of lysosomal stress on DPSC-derived Schwann cell behavior using chloroquine, a lysosomal stress-inducing agent [10]. While the exact effect of chloroquine on the lysosomal–autophagic pathway remains under debate, it is believed to accumulate in the lysosomal lumen, where it is protonated, preventing its escape from the lysosomal system. By sequestering protons, chloroquine disrupts lysosomal acidification and inhibits lysosomal–autophagic fusion [39,40,41], leading to lysosomal dysfunction, overload, and eventually lysosomal stress. Chloroquine is frequently used as a positive control for lysosomal damage, particularly in the context of lysosomal storage disorders [39,40,42].

Our data confirm that chloroquine influences lysosomal behavior in DPSC-derived Schwann cells, evidenced by a significant increase in the Lysotracker signal. Notably, the lowest concentration of chloroquine resulted in the highest Lysotracker signal. This observation may be explained by the hydrophobic weak base nature of Lysotracker, which is attracted to the acidic environment of lysosomes [43], while chloroquine acts as an alkalizing agent, raising the lysosomal pH and potentially reducing the Lysotracker accumulation [39]. Hence, high concentrations of chloroquine increase the lysosomal pH and reduce the Lysotracker accumulation in the lysosomes. This hypothesis could be tested by performing a pH-independent assay after exposure to higher (>20 µM) chloroquine concentrations, for example a LAMP1 staining.

On the other hand, previous research demonstrated that low chloroquine concentrations inhibit autophagy, while high chloroquine concentrations cause lysosomal permeability [44], which would be another plausible explanation for the loss of the Lysotracker signal in a higher or prolonged chloroquine concentration and incubation, respectively. Moreover, this phenomenon has been used as a method to study lysosomal permeability [30,45,46]. The TEM and a galectin-3-LAMP1 puncta assay confirmed an increase in the lysosomal size and permeability in human DPSC-derived Schwann cells after prolonged chloroquine treatment. However, caution is warranted when using galectin 3-LAMP1 staining in the context of the PNS, as recent data indicate an endogenous galectin 3 upregulation in dedifferentiated Schwann cells, which is relevant for peripheral neuropathies [47]. 

Lysosomal permeability, implicated as a risk factor for lysosome-induced apoptosis, activates the inflammasome response, leading to caspase activation and eventually apoptosis [48], which was also demonstrated in other cell types [49,50,51,52]. Our assays demonstrated reduced viability in DPSC-derived Schwann cells after 5 h of chloroquine incubation, with apoptosis becoming visible after 20 h as demonstrated by a live Caspase-3 staining. Prolonged exposure to chloroquine is likely to induce Schwann cell apoptosis, possibly due to increased permeabilization of the membrane [31]. Nevertheless, lysosomal permeability has also been reported to induce caspase-independent cell death [30]. Therefore, further investigation into the precise underlying mechanisms leading to cell death is needed.

In addition, DPSC-derived Schwann cells are cultured in a growth factor-enriched medium supplemented with forskolin, an activator of adenylate cyclase. Moreover, during the initiation of differentiation, the cells are cultured with retinoic acid, a neural morphogen. Both forskolin and retinoic acid are known to affect the lysosomal pH and function [53,54,55,56]. This could potentially induce a state of hyper-acidification, which might influence the observed effects on lysosomal stress [53,54,55]. For this reason, including proper control groups is imperative.

Importantly, while chloroquine is commonly used as a positive control for lysosomal dysfunction, stress, and permeability, its multifaceted roles, including anti-inflammatory and immunomodulatory effects, should be considered. Moreover, chloroquine reduces calcium signaling and the activity of matrix metalloproteinases (MMPs) [57,58,59]. Recently, chloroquine has also been reported to be an allosteric inhibitor of the proteasomal degradation system and is known to induce neuronal cell death, despite its neuroprotective functions [60,61,62]. In the context of peripheral neurodegenerative diseases, the suitability of chloroquine as a positive control may be questioned due to its reported impact on various cellular functions [60,61,62]. 

Moreover, in future experiments, it might be beneficial to compare our chemically induced lysosomal dysfunction model with DPSC-derived Schwann cells isolated from patients with genetic forms of neurodegenerative diseases in which Schwann cells play an important role, such as the Charcot–Marie–Tooth disease type 1 [7,36].

## 5. Conclusions

In summary, our findings present a chemically induced model that demonstrates lysosomal dysfunction and stress in DPSC-derived Schwann cells, which are more readily accessible compared to primary Schwann cells [20,21]. This could potentially pave the way for future research into the implications of lysosomal dysfunction in Schwann cells for myelinating disorders of the peripheral nervous system.

## Data Availability

All data supporting the findings of this study are available from the corresponding author on reasonable request.
